# Kaempferol attenuates nonalcoholic steatohepatitis by regulating serum and liver bile acid metabolism

**DOI:** 10.3389/fphar.2022.946360

**Published:** 2022-09-29

**Authors:** Yifei Lu, Mingmei Shao, Caiyun Zhang, Hongjiao Xiang, Junmin Wang, Tao Wu, Guang Ji

**Affiliations:** ^1^ Institute of Interdisciplinary Integrative Medicine Research, Shanghai University of Traditional Chinese Medicine, Shanghai, China; ^2^ Yueyang Hospital of Integrated Traditional Chinese and Western Medicine, Shanghai University of Traditional Chinese Medicine, Shanghai, China; ^3^ Baoshan District Hospital of Intergrated Traditional Chinese and Western Medicine, Shanghai, China; ^4^ Institute of Digestive Disease, Longhua Hospital, Shanghai University of Traditional Chinese Medicine, Shanghai, China

**Keywords:** kaempferol, NASH, bile acid, metabolomics, metabolism

## Abstract

**Objective:** Changes in bile acids (BAs) are increasingly recognized as potential targets for non-alcoholic steatohepatitis (NASH). Kaempferol has been proved to be anti-inflammatory and reduce the disorder of lipid metabolism. In order to analyze the BA profile in NASH mice and determine the predictive biomarkers of kaempferol treatment, serum-targeted metabolomics and liver tissue RNA sequencing (RNA-seq) were carried out.

**Design:** Six normal control mice (NC group), eight HFD-fed mice (HFD group), and eight kaempferol-treated HFD-fed mice (HFD + KP group) were included in the present study. Ultra-performance liquid chromatography coupled to a tandem mass spectrometry system (UPLC-MS/MS) was used to quantify serum and liver BAs, and RNA-seq was used to quantify liver differentially expressed genes related to BA metabolism.

**Results:** The serum levels of CA, βMCA, UDCA, and 12-DHCA, as well as ωMCA in both the serum and liver, were significantly decreased in the HFD group compared with those in the NC group, and kaempferol can increase the serum levels of βMCA, UDCA, and ωMCA and the liver level of 12-DHCA. The serum levels of TDCA, THDCA, TUDCA, TDCA/CA, and TDCA/DCA were significantly increased in the HFD group compared with those of the NC group, and kaempferol can decrease them. Furthermore, NASH mice had a higher liver level of total CA%, total CDCA%, primary BAs/secondary BAs, 12α-OH BAs/non-12α-OH Bas, and conjugated BAs/unconjugated BAs, and all decreased after kaempferol treatment. According to the RNA-seq results, we found that compared with the NC group, the mRNA expression of cholesterol-7α-hydroxylase (CYP7A1) in the HFD group was significantly increased, and the mRNA expression of sterol 12α‐hydroxylase (CYP8B1) and multidrug resistance-related protein 3 (MRP3) was significantly decreased, while kaempferol significantly promoted the mRNA expression of mitochondrial sterol 27-hydroxylase (CYP27A1) and Na^+^ -taurocholate cotransporting polypeptide (NTCP).

**Conclusion:** βMCA, CA, UDCA, 12-DHCA, ωMCA, CDCA, TωMCA, TDCA, THDCA, TCDCA, and TUDCA in the serum, as well as 6,7-diketoLCA, 12-DHCA, and ωMCA in the liver, may be potential biomarkers for kaempferol to improve NASH. HFD-induced NASH may be associated with the increase of CYP7A1 and the decrease of CYP8B1, leading to increased BA synthesis, and the decrease of MRP3 leading to decreased BA synthesis, and kaempferol may alleviate NASH by increasing CYP27A1 and NTCP to enhance BA transport.

## Introduction

As a common liver disease, non-alcoholic steatohepatitis (NASH) is often characterized by inflammatory cell infiltration and lipid droplets in more than 5% of hepatocytes (Younossi et al., 2016). NASH has already become increasingly common globally, and the incidence of NASH is increasing year by year. Studies have predicted that by 2030, the prevalence of NASH cases will increase by 63% (Tawfiq et al., 2022), and its pathogenesis may be related to insulin resistance, oxidative stress, and endoplasmic reticulum stress (ERS) caused by excessive fat accumulation, resulting in lipotoxic liver injury (Machado and Diehl, 2016), yet the exact mechanism remains unclear.

Bile acids (BAs) stored in bile are synthesized from cholesterol in the liver and released into the small intestine. Primary BAs including cholic acid (CA) are initiated by cholesterol 7α-hydroxylase (CYP7A1), and then, sterol-12α-hydroxylase (CYP8B1) (Chiang, 2009) and muricholic acids (MCAs) are initiated by sterol-27-hydroxylase (CYP27A1) (Wahlström et al., 2016). In the gut, secondary BAs are deconjugated by bacterial enzymes including lithocholic acid (LCA) and deoxycholic acid (DCA). It is then reabsorbed through portal blood flow and carried back to the liver. Studies have shown that the level and composition of BA are related to metabolic diseases such as dyslipidemia (Chen et al., 2020). Moreover, many studies have shown that there are significant differences in BA levels between patients with NASH and normal people (Jiao et al., 2022).

Kaempferol (KP), a biologically active flavonol, has been shown to have broad pharmacological effects on inflammation (Alshehri et al., 2022), oxidation, and regulation of tumors (Tie et al., 2021). Our previous studies have confirmed that kaempferol mainly reduced inflammation and lipid deposition through the liver X receptor (LXR)-lysophosphatidylcholine acyltransferase 3-ERS pathway to improve NASH (Xiang et al., 2021). In order to fully understand the mechanism of action of KP and provide new ideas for its clinical application in the treatment of liver diseases, our research group has also discovered the potential targets that kaempferol may regulate through transcriptomics and metabolomics (Lu et al., 2020). The purpose of this article is to find the potential biomarker of kaempferol based on our previously successfully established NASH mouse model in regulating BAs and hope to find a new direction to the treatment of NASH.

In order to explore the characteristics and differences of BA, we collected liver issue and serum of healthy mice, as well as NASH mice and NASH mice treated with kaempferol. BA profiles were analyzed in these samples. Here, we intend to present the distinct BA profiles and genes related to BA metabolism of NASH and NASH treatments and to compare the similarities and differences of BA compositions among three groups.

## Materials and methods

### Animals and treatment

Specific-pathogen-free male C57BL/6J mice (6-week-old) were purchased from Shanghai SLAC Laboratory (Shanghai, China). Seven normal control mice (NC group), eight HFD-fed mice (HFD, 60.0% fat, 20.0% carbohydrate, and 20.0% protein; D12492, Research Diets), and eight kaempferol-treated HFD-fed mice (HFD + KP, HFD diet supplemented with kaempferol, a dose of 0.5 ml/100 g for last 4 weeks) were included by using a completely randomized design based on body weight and were fed with water ad libitum for 24 weeks to induce metabolic disorders and fatty liver. The specific feeding methods and the drug proportion of kaempferol are described in detail in our previous study (Lu et al., 2020).

### Sample preparation and bile acid analysis

All mice’s blood were centrifuged (3000 r/min, 15 min, 4°C), and serum and 50 mg tissue were stored at −80°C refrigerator for further analysis. Quantitative assessment of BAs is performed by laboratory-developed test kit-Metabolite Array (HMI, Shenzhen, Guangdong, China), according to previously reported methods (Lu et al., 2020; Wu et al., 2021). BAs were quantified by ultra-performance liquid chromatography coupled to a tandem mass spectrometry system (UPLC/MS/MS, ACQUITY UPLC-Xevo TQ-S, Waters Corp, Milford, MA, United States). The specific analysis and experimental methods are shown in the supplementary material.

### RNA sequence

Total RNA (1 μg) was used for removing the rRNAs using Ribo-Zero rRNA Removal kits (Illumina, San Diego, CA, United States), and RNA libraries were constructed by using rRNA-depleted RNAs with the TruSeq Stranded total RNA Library Prep Kit (Illumina, San Diego, CA, United States) and qualified and quantified using the BioAnalyzer 2100 system (Agilent Technologies, Inc., United States). Then, 10 p.m. libraries were denatured as single-stranded DNA molecules, captured on Illumina flow cells, amplified *in situ* as clusters, and finally sequenced for 150 cycles on the Illumina HiSeq Sequencer, according to the manufacturer’s instructions. More detailed experimental details have been presented in the previous study (GEO number: GSE145665) (Lu et al., 2020).

### Statistical analysis

The data were presented as mean ± SEM. Two-tailed Student’s t-test and one-way analysis of variance (ANOVA) with Tukey’s test were used. Metabolomics analysis was analyzed with principal components analysis (PCA) and partial least squares–discriminant analysis (PLS-DA). GraphPad Prism software 8.0 (La Jolla, CA) was used to analyze the data.

## Results

### Quantitative detection of BAs

Previous targeted metabolomics protocols and profiling protocols (Lu et al., 2020) were used to quantify BAs in serum and liver among three groups. Briefly, this study was designed to assess bile acids in the study samples using laboratory-developed test (LDT) kit – Metabolite Array (HMI, Shenzhen, Guangdong, China). The test was performed on an UPLC-MS/MS system for linearity, carry-over, matrix effects, accuracy, and precision and stability. The software IP4M can perform a collection of data processing, interpretation, and visualization.

### Multivariate data analysis based on serum BA profiles and potential metabolite identification among control, NASH models, and kaempferol groups

**FIGURE 1:**
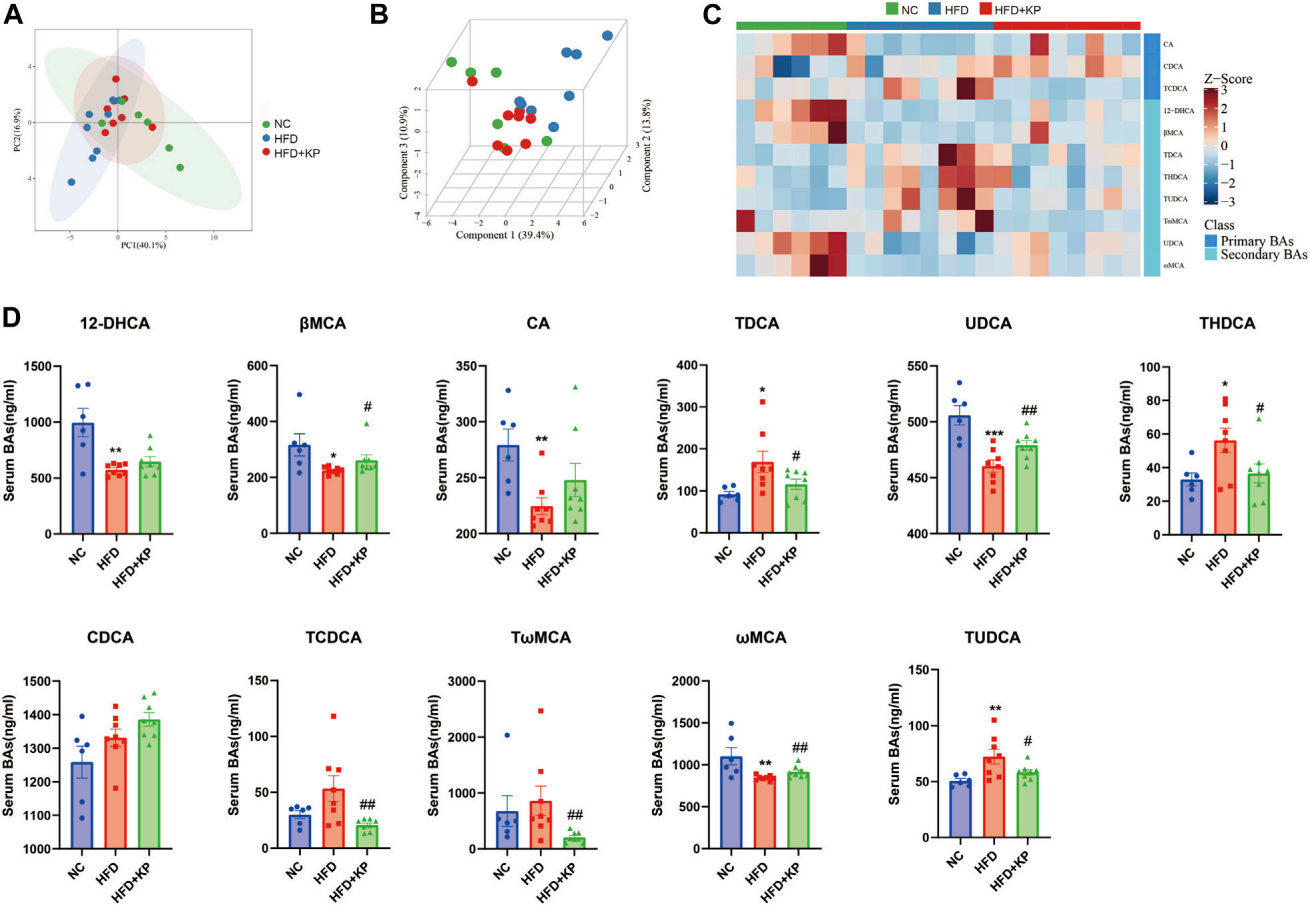
Comparison of the serum BA level and composition among three groups. **(A)** PCA score plots show separation among three groups based on 19 serum bile acids. **(B)** PLS-DA score plots show clear separation among three groups based on 19 serum bile acids. **(C)** Heatmap of 11 serum bile acid biomarkers among three groups. **(D)** Differential serum bile acids significantly differed among three groups. All values were expressed as mean ± SEM. *p < 0.05, **p < 0.01, and ***p < 0.001, when NASH models (HFD group) were compared to control groups (NC group); #*p* < 0.05 and ##*p* < 0.01, when NASH models (HFD group) were compared to NASH models treated with kaempferol (the HFD + KP group).

The NASH mouse model was successfully based on the protocols established by our previous study (Lu et al., 2020). We found that NASH mice have the characteristics of high body weight, elevated serum levels of ALT, AST, TC, LDL, and liver TG levels, as well as obvious steatosis and inflammatory cell infiltration. Kaempferol can improve NASH in both serology and liver histology. KP uniformizes the group, diminishing the variance between group samples from PCA and PLS-DA models established with the identified serum BAs. The UPLC-MS/MS spectra were visualized through the PCA to determine the actual clusters (Figure 4A). We observed a clear separation between the NC group and HFD group mice from the PLS-DA model established with the identified serum BAs. Similarly, a separation trend between the HFD group and HFD + KP group was also observed (Figure 1B). A total of 19 serum BA metabolites were quantitatively determined in the present study, and the detailed information is shown in Table 1 and Supplementary Figure S1A, B. The THDCA and TUDCA concentrations were found significantly higher in the sera of NASH mice than in controls. The 12-DHCA, βMCA, CA, UDCA, and ωMCA concentrations were found significantly lower in the sera of NASH mice than in controls. The βMCA, UDCA and ωMCA concentrations were found significantly higher in the sera of NASH mice treated by kaempferol than in NASH mice. The serum TDCA, THDCA, TCDCA, TωMCA and TUDCA concentrations were found significantly lower NASH mice treated by kaempferol than in NASH mice (Table 1). The metabolites of variable influence on projection (VIP) value > 1 and *p* < 0.05, |log2FC| ≥ 0 were selected to obtain the differential metabolites. Finally, 11 potential serum biomarkers, namely, βMCA, CA, UDCA, 12-DHCA, ωMCA, CDCA, TωMCA, TDCA, THDCA, TCDCA, and TUDCA, were found (Figure 1C), and interestingly, most of the differential serum BA metabolites belong to secondary BAs (Supplementary Figure S1B). We found that serum levels of UDCA, ωMCA, CA, βMCA, and 12-DHCA were significantly decreased in the HFD group, and furthermore, kaempferol can increase the serum level of UDCA, ωMCA, and βMCA. In addition, serum levels of TDCA, TUDCA, and THDCA were increased in the HFD group and decreased in the HFD + KP group compared with those in the HFD group. Serum levels of TωMCA and TCDCA were also found to be decreased after treatment by kaempferol. However, there were no significant differences in serum CDCA among the three groups (Figure 1D).

**TABLE 1:** Serum BA levels in different groups.

Variable (ng/ml)	NC (n = 6)	HFD (n = 8)	HFD + KP (n = 8)
TωMCA	677.5 ± 276.3	864.1 ± 263.1	208.9 ± 33.59^##^
TUDCA	50.67 ± 2.044	72.38 ± 6.541^**^	58.25 ± 2.520^#^
THDCA	33 ± 3.907	56.25 ± 7.176^*^	36.63 ± 5.666^#^
TCA	787.3 ± 215.6	592.4 ± 111.0	323.5 ± 62.48^#^
TDCA	91.67 ± 6.697	169 ± 25.14^*^	115.8 ± 12.39^#^
TCDCA	30 ± 3.587	53.38 ± 11.51	20.75 ± 1.820^##^
12-DHCA	995.8 ± 127.0	575.9 ± 17.90^**^	647.0 ± 43.29
ωMCA	1103 ± 102.6	845.4 ± 10.38^**^	916.9 ± 24.56^##^
βMCA	316 ± 39.61	224.6 ± 4.563^*^	261.1 ± 19.30^#^
βUDCA	107.8 ± 5.319	113.4 ± 0.6250	109.4 ± 3.914
UDCA	505.8 ± 8.765	460.5 ± 5.268^***^	479.0 ± 4.217^##^
HDCA	46 ± 10.24	18.75 ± 2.541^*^	26.88 ± 4.510
CA	279.2 ± 14.28	224.6 ± 7.535^**^	248.0 ± 14.81
GCA	15 ± 1.713	26.5 ± 3.698*	22.5 ± 3.694
CDCA	1259 ± 47.77	1332 ± 25.68	1386 ± 19.96
GCDCA	50.5 ± 9.577	34.25 ± 3.922	46 ± 5.120
GDCA	100.3 ± 6.386	95.63 ± 2.008	98.88 ± 2.503
LCA	885 ± 45.25	906.9 ± 46.73	902.5 ± 34.58
DCA	6587.667 ± 410.2	6066.5 ± 266.2	5864.375 ± 108.6

Note: All values were expressed as mean ± SEM. *p < 0.05, **p < 0.01, and ***p < 0.001, when NASH models (the HFD group) were compared to control groups (the NC group); #*p* < 0.05 and ##*p* < 0.01, when NASH models (the HFD group) were compared to NASH models treated with kaempferol (the HFD + KP group).

### Serum BA ratios and percentage altered significantly among three groups

**FIGURE 2:**
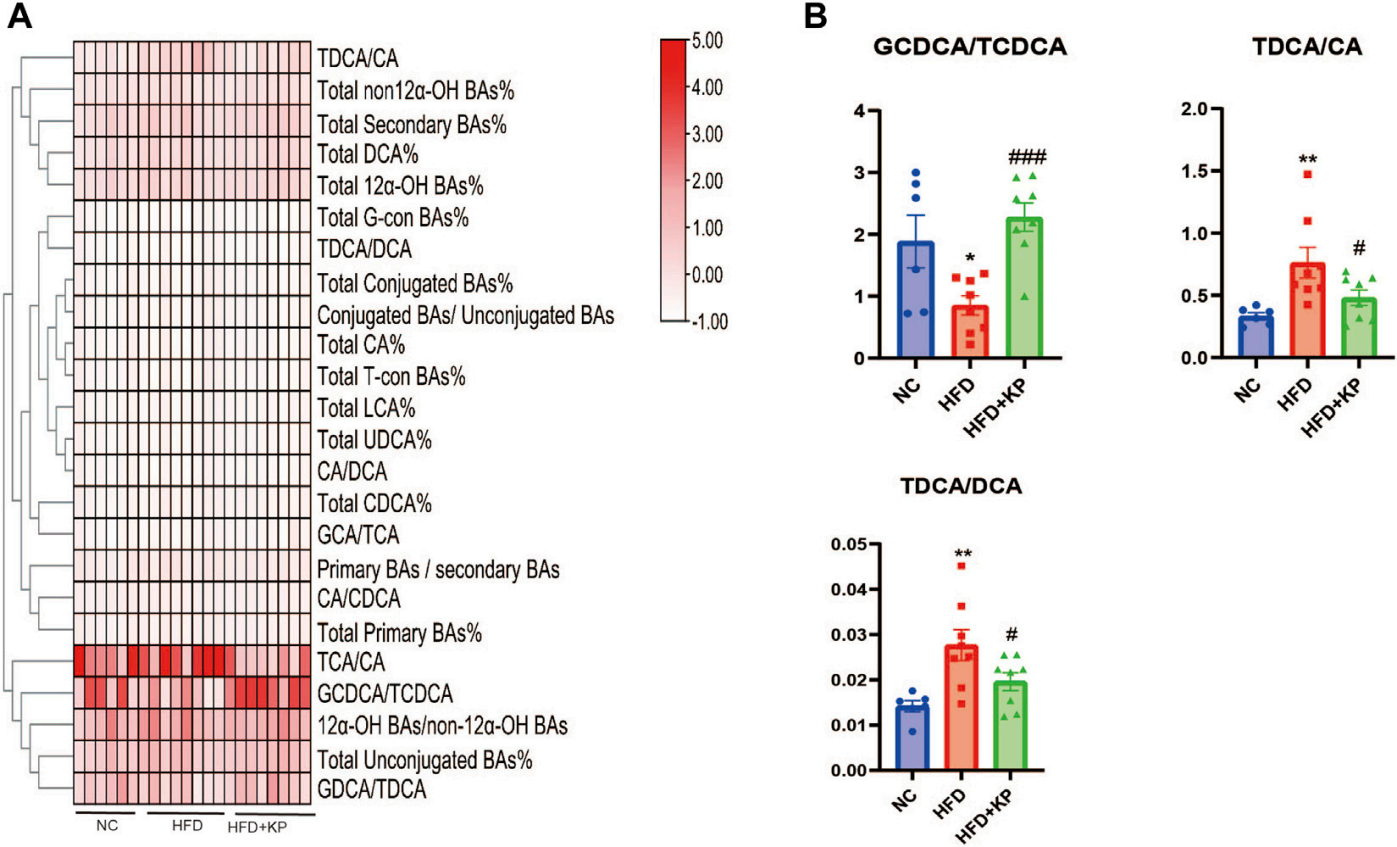
Comparison of serum BA ratios and percentage among three groups. **(A)** Heatmap of serum BA ratios and percentage among three groups. **(B)** Differential serum BA ratios significantly differed among three groups. All values were expressed as mean ± SEM. *p < 0.05 and **p < 0.01, when NASH models (the HFD group) were compared to control groups (NC group); #*p* < 0.05, ##*p* < 0.01, and ###*p* < 0.001, when NASH models (the HFD group) were compared to NASH models treated with kaempferol (the HFD + KP group).

The serum BA ratios and percentage are calculated and summarized in Table 2 to further study the significance of BA profiles among three groups (Figure 2A). The level of GCDCA/TCDCA was much lower in HFD group than that in control groups, while it was increased in the HFD + KP group compared with that in the HFD group. In addition, the levels of TDCA/CA and TDCA/DCA were increased in HFD group than in control groups, while decreased in the HFD + KP group compared with those in the HFD group (Figure 2B).

**TABLE 2:** Serum BA ratios and percentage altered significantly among three groups.

Variable	NC (n = 6)	HFD (n = 8)	HFD + KP (n = 8)
Total CA (ng/ml)	1082 ± 211.7	843.5 ± 113.9	594.0 ± 63.49
Total CA%	7.75 ± 1.40	6.51 ± 0.72	5.01 ± 0.49
Total CDCA (ng/ml)	1339 ± 43.83	1419 ± 32.31	1453 ± 21.68
Total CDCA%	9.71 ± 0.54	11.25 ± 0.44	12.35 ± 0.12
CA/CDCA	0.22 ± 0.02	0.17 ± 0.005	0.18 ± 0.009
Total UDCA (ng/ml)	602.5 ± 16.44	551.6 ± 6.70	564.1 ± 8.95
Total UDCA%	0.05 ± 0.002	0.05 ± 0.002	0.06 ± 0.0007
Total DCA (ng/ml)	6780 ± 414.6	6331 ± 279.2	6079 ± 117.2
Total DCA%	0.49 ± 0.02	0.50 ± 0.02	0.52 ± 0.009
Total LCA (ng/ml)	885 ± 45.25	906.9 ± 46.73	902.5 ± 34.58
Total LCA%	0.06 ± 0.004	0.07 ± 0.003	0.08 ± 0.003
Total primary BAs (ng/ml)	2421 ± 231.1	2263 ± 138.2	2047 ± 76.27
Total primary BAs%	0.17 ± 0.02	0.18 ± 0.008	0.17 ± 0.004
Total secondary BAs (ng/ml)	8160 ± 452.6	7655 ± 302.1	7424 ± 123.2
Total secondary BAs%	0.59 ± 0.02	0.60 ± 0.02	0.63 ± 0.009
Primary BAs/secondary BAs	0.30 ± 0.04	0.30 ± 0.02	0.28 ± 0.01
Total 12α-OH BAs (ng/ml)	7861 ± 362.2	7175 ± 321.9	6673 ± 148.8
Total 12α-OH BAs%	0.56 ± 0.006	0.56 ± 0.01	0.57 ± 0.008
Total non-12α-OH BAs (ng/ml)	6060 ± 258.8	5557 ± 279.7	5099 ± 133.6
Total non-12α-OH BAs%	0.45 ± 0.006	0.44 ± 0.001	0.43 ± 0.008
12α-OH BAs/non-12α-OH BAs	1.30 ± 0.03	1.30 ± 0.06	1.32 ± 0.04
Total conjugated BAs (ng/ml)	1126 ± 213.2	1044 ± 145.7	685.6 ± 74.81
Total conjugated BAs%	0.08 ± 0.01	0.08 ± 0.009	0.06 ± 0.006
Total unconjugated BAs (ng/ml)	12796 ± 591.1	11688 ± 417.1	11087 ± 152.4
Total unconjugated BAs%	0.92 ± 0.01	0.92 ± 0.009	0.94 ± 0.006
Conjugated BAs/unconjugated BAs	0.09 ± 0.02	0.008 ± 0.01	0.06 ± 0.007
Total T-con BAs (ng/ml)	959.7 ± 218.0	887.1 ± 141.9	518.3 ± 70.52
Total T-con BAs%	0.07 ± 0.01	0.07 ± 0.009	0.04 ± 0.006
Total G-con BAs (ng/ml)	165.8 ± 14.06	156.4 ± 5.80	167.4 ± 8.87
Total G-con BAs%	0.012 ± 0.0009	0.01 ± 0.0006	0.01 ± 0.0006
T-con/G-con BA	6.15 ± 1.53	5.58 ± 0.80	3.08 ± 0.36
CA/DCA	0.04 ± 0.002	0.04 ± 0.002	0.04 ± 0.003
TDCA/DCA	0.01 ± 0.001	0.03 ± 0.003**	0.020 ± 0.002#
TDCA/CA	0.33 ± 0.03	0.76 ± 0.12**	0.48 ± 0.06#
TCA/CA	2.93 ± 0.90	2.66 ± 0.51	1.35 ± 0.30
GCA/TCA	0.03 ± 0.009	0.06 ± 0.01	0.09 ± 0.02
GDCA/TDCA	1.41 ± 0.15	0.64 ± 0.08	0.93 ± 0.11
GCDCA/TCDCA	1.88 ± 0.43	0.85 ± 0.16*	2.28 ± 0.23###

Note: All values were expressed as mean ± SEM. *p < 0.05 and **p < 0.01, when NASH models (the HFD group) were compared to control groups (the NC group); #*p* < 0.05, ###*p* < 0.001, when NASH models (the HFD group) were compared to NASH models treated with kaempferol (the HFD + KP group).

### Correlations between serum BA-relevant variables and clinical indices

**FIGURE 3:**
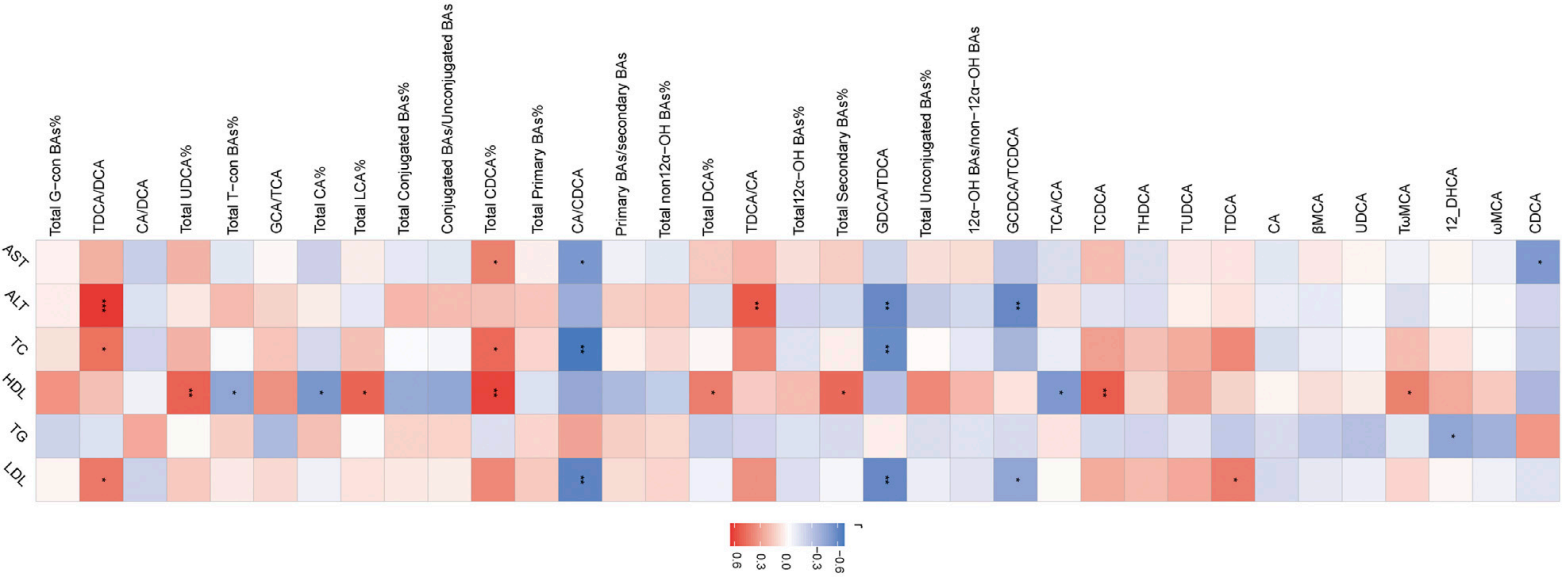
Heatmap of Spearman correlation coefficients between serum BAs and serum bio-chemical parameters from all samples in the three groups. n = 22, six samples for control group, and eight samples for the other two groups. The gradient colors represent the correlation coefficients, with red color being more positive and blue color being more negative. *p < 0.05, **p < 0.01, and ***p < 0.001 (Spearman’s correlation with the *post hoc* correction using the Holm method).

The association of serum BAs and serum biochemical parameters was performed among three groups by Spearman correlation analysis. We found that CDCA and CA/CDCA were significantly negative, while total CDCA% had a positive correlation with serum AST. TDCA/DCA and TDCA/CA had a significantly positive correlation, while GDCA/TDCA and GCDCA/TCDCA had a negative correlation with serum ALT. TDCA/DCA and total CDCA% had a positive correlation, while CA/CDCA and GDCA/TDCA had a negative correlation with serum LDL. GDCA/TDCA and CA/CDCA had a negative correlation, and total CDCA% and TDCA/DCA had a positive correlation with serum TC. TCA/CA, total CA%, and total-con BA% had a negative correlation and TωMCA, TCDCA, total secondary BA%, total DCA%, total CDCA%, total LCA%, and total UDCA% had a positive correlation with serum HDL. Interestingly, only 12-DHCA had a negative correlation with serum TG (Figure 3).

### Multivariate data analysis based on liver BA profiles and potential metabolite identification among control, NASH models, and kaempferol groups

**FIGURE 4:**
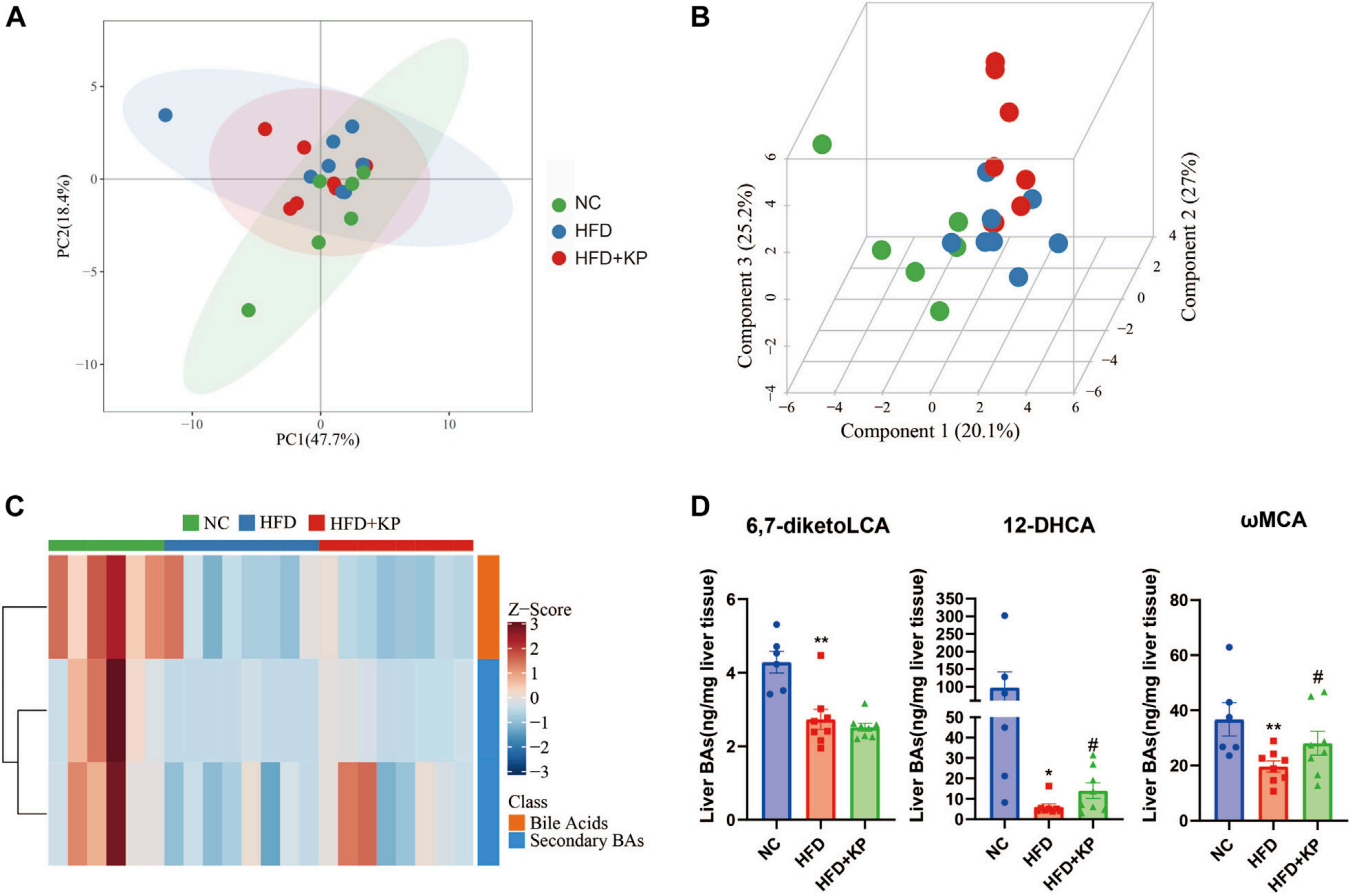
Comparison of the liver BA level and composition among three groups. **(A)** PCA score plots show separation among three groups based on 28 liver bile acids. **(B)** PLS-DA score plots show clear separation among three groups based on 28 liver bile acids. **(C)** Heatmap of three liver bile acid biomarkers among three groups. **(D)** Differential liver bile acids significantly differed among three groups. All values were expressed as mean ± SEM. *p < 0.05 and **p < 0.01, when NASH models (the HFD group) were compared to control groups (the NC group); #*p* < 0.05, when NASH models (the HFD group) were compared to NASH models treated with kaempferol (the HFD + KP group).

The UPLC-MS/MS spectra were visualized through the PCA to determine the actual clusters (Figure 1A). We observed a clear separation between the NC group and HFD group mice from the PLS-DA model established with the identified serum BAs. Similarly, a separation trend between the HFD group and HFD + KP group was also observed (Figure 4B). Twenty-eight serum BA metabolites were quantitatively determined in the present study, and the detailed information is shown in Table 3 and Supplementary Figure S2A, B. Interestingly, most of the differential liver BA metabolites belong to primary BAs (Supplementary Figure S2B). The 6,7-diketoLCA,12-DHCA and ωMCA concentrations were found significantly lower in the sera of NASH mice than in controls. The 12-DHCA and ωMCA concentrations were found significantly higher in the sera of NASH mice treated by kaempferol than NASH mice (Table 3). The metabolites of VIP value > 1 and *p* < 0.05, |log2FC| ≥ 0 were selected to obtain the differential metabolites. Finally, three potential liver biomarkers were 6,7-diketoLCA, 12-DHCA, and ωMCA (Figure 4C). We found that all the three liver BAs were significantly decreased in the HFD group, and furthermore, kaempferol increased the liver levels of 12-DHCA and ωMCA (Figure 4D).

**TABLE 3:** Liver BA levels in different groups.

Variable (ng/mg liver tissue)	NC (n = 6)	HFD (n = 8)	HFD + KP (n = 8)
TCDCA	6.91 ± 2.30	39.13 ± 22.91	19.27 ± 5.90
TDCA	14.41 ± 4.34	42.64 ± 27.08	33.91 ± 12.60
GHCA	0.33 ± 0.08	0.73 ± 0.38	0.60 ± 0.20
GDCA	0.06 ± 0.01	0.06 ± 0.01	0.06 ± 0.01
TCA	171.9 ± 50.95	280.6 ± 88.38	243.0 ± 55.97
LCA	0.54 ± 0.03	0.50 ± 0.02	0.50 ± 0.02
3-DHCA	2.06 ± 0.08	2.14 ± 0.16	2.32 ± 0.14
DCA	1.24 ± 0.21	1.18 ± 0.10	1.23 ± 0.10
CDCA	2.65 ± 0.37	3.47 ± 0.51	2.95 ± 0.33
CDCA-24G	1.13 ± 0.06	1.11 ± 0.06	1.06 ± 0.03
NorDCA	1.51 ± 0.14	1.36 ± 0.06	1.36 ± 0.06
apoCA	0.18 ± 0.02	0.15 ± 0.02	0.18 ± 0.02
7-KetoDCA	1.14 ± 0.28	0.89 ± 0.04	0.89 ± 0.04
UDCA	5.34 ± 0.82	4.79 ± 0.38	6.40 ± 0.74
HDCA	4.56 ± 1.04	5.34 ± 1.00	6.03 ± 0.90
6,7-DiketoLCA	4.29 ± 0.30	2.73 ± 0.28*	2.52 ± 0.11
CA	5.97 ± 1.83	5.66 ± 1.83	5.54 ± 1.67
βCA	5.91 ± 1.66	5.99 ± 1.68	6.54 ± 1.27
7-KetoLCA	4.19 ± 1.13	4.41 ± 1.22	4.85 ± 0.96
12-KetoLCA	3.86 ± 0.72	2.48 ± 0.44	2.18 ± 0.24
NorCA	0.43 ± 0.09	0.48 ± 0.11	0.49 ± 0.04
GCA	21.60 ± 7.42	65.02 ± 34.47	40.89 ± 15.82
ωMCA	36.72 ± 6.05	19.65 ± 2.08**	28.10 ± 4.32#
βMCA	16.02 ± 5.14	24.28 ± 5.71	21.63 ± 3.77
12-DHCA	97.47 ± 44.48	6.06 ± 1.47*	14.03 ± 3.82#
7,12-DiketoLCA	1.84 ± 0.18	1.70 ± 0.11	1.73 ± 0.11
THDCA	10.45 ± 4.36	20.11 ± 11.45	22.40 ± 8.23
TωMCA	52.31 ± 24.88	77.16 ± 47.88	55.81 ± 22.78

Note: All values were expressed as mean ± SEM. *p < 0.05, **p < 0.01, when NASH models (the HFD group) were compared to control groups (the NC group); #*p* < 0.05, when NASH models (the HFD group) were compared to NASH models treated with kaempferol (the HFD + KP group).

### Liver BA ratios and percentage altered significantly among three groups

**FIGURE 5:**
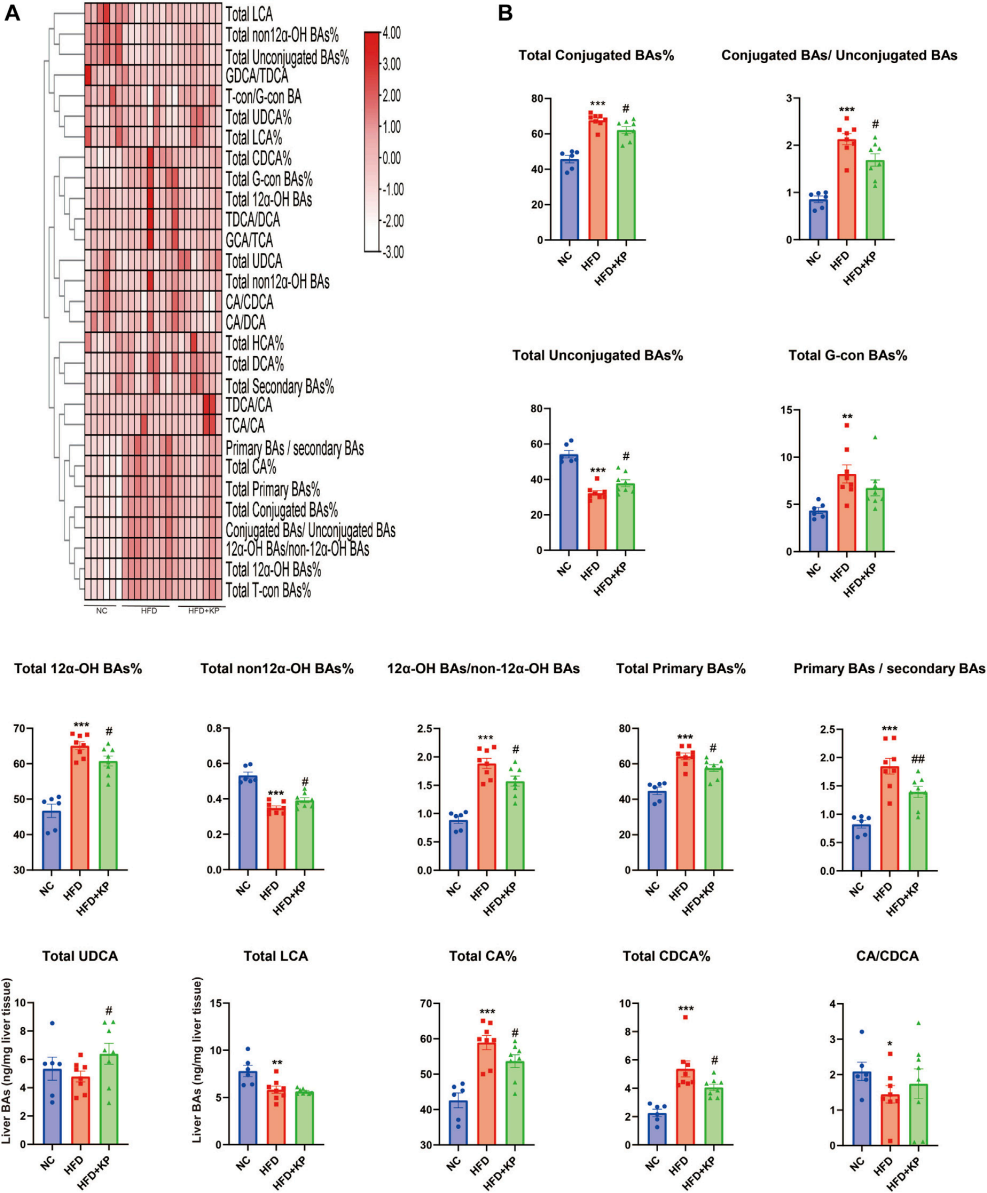
Comparison of liver BA ratios and percentage among three groups. **(A)** Heatmap of liver BA ratios and percentage among three groups. **(B)** Differential liver BA ratios significantly differed among three groups. All values were expressed as mean ± SEM. *p < 0.05, **p < 0.01, and ***p < 0.001, when NASH models (HFD group) were compared to control groups (NC group); #*p* < 0.05 and ##*p* < 0.01, when NASH models (HFD group) were compared to NASH models treated with kaempferol (the HFD + KP group).

The liver BA ratios and percentage are summarized in Table 4 to further study the significance of BA profiles among three groups (Figure 5A). The levels of the total conjugated BAs%, conjugated BAs/unconjugated BAs, total 12α-OH BAs%, 12α-OH BAs/non-12α-OH BAs, total primary BAs%, primary BAs/secondary BAs, total CA%, and total CDCA% were much higher in HFD groups than those in control groups, while decreased in the HFD + KP group compared with that in the HFD group. In addition, the levels of total unconjugated BAs% and total non-12α-OH BAs were decreased in HFD groups than in control groups, while increased in the HFD + KP group compared with those in the HFD group (Figure 5B).

**TABLE 4:** Liver BA ratios and percentage altered significantly among three groups.

Variable	NC (n = 6)	HFD (n = 8)	HFD + KP (n = 8)
Total CA (ng/mg liver tissue)	199.7 ± 59.73	351.4 ± 124.0	289.6 ± 71.57
Total CA%	42.61 ± 2.10	58.95 ± 1.99***	53.70 ± 1.80#
Total CDCA (ng/mg liver tissue)	9.56 ± 2.66	42.60 ± 23.40	22.22 ± 6.10
Total CDCA%	2.26 ± 0.26	5.37 ± 0.56***	4.06 ± 0.22#
CA/CDCA	2.09 ± 0259	1.45 ± 0.25*	1.74 ± 0.42
Total UDCA (ng/mg liver tissue)	5.34 ± 0.82	4.79 ± 0.36	6.40 ± 0.74#
Total UDCA%	1.55 ± 0.29	1.31 ± 0.24	1.66 ± 0.26
Total DCA (ng/mg liver tissue)	17.21 ± 4.54	45.23 ± 27.17	36.56 ± 12.61
Total DCA%	4.08 ± 0.47	6.10 ± 1.13	7.01 ± 0.76
Total LCA (ng/mg liver tissue)	7.81 ± 0.60	5.82 ± 0.38*	5.65 ± 0.10
Total LCA%	2.76 ± 0.86	1.81 ± 0.48	1.86 ± 0.55
Total HCA (ng/mg liver tissue)	0.33 ± 0.08	0.73 ± 0.38	0.60 ± 0.20
Total HCA%	0.10 ± 0.03	0.11 ± 0.02	0.13 ± 0.03
Total primary BAs (ng/mg liver tissue)	209.1 ± 62.01	393.9 ± 147.0	311.7 ± 77.59
Total primary BAs%	44.81 ± 2.16	64.26 ± 1.85***	57.70 ± 1.86#
Total secondary BAs (ng/mg liver tissue)	31.46 ± 6.86	58.14 ± 29.09	51.08 ± 13.38
Total secondary BAs%	8.26 ± 1.29	9.20 ± 1.31	10.78 ± 1.10
Primary BAs/secondary BAs	0.82 ± 0.07	1.85 ± 0.14***	1.39 ± 0.10##
Total 12α-OH BAs (ng/mg liver tissue)	217.1 ± 64.17	397.0 ± 149.9	326.5 ± 82.99
Total 12α-OH BAs%	46.74 ± 1.89	65.11 ± 1.11***	60.79 ± 1.40#
Total non-12α-OH BAs (ng/mg liver tissue)	257.9 ± 90.36	222.8 ± 95.67	200.0 ± 47.37
Total non-12α-OH BAs%	0.53 ± 0.02	0.35 ± 0.01***	0.39 ± 0.01#
12α-OH BAs/non-12α-OH BAs	0.89 ± 0.06	1.89 ± 0.09***	1.57 ± 0.09#
Total conjugated BAs (ng/mg liver tissue)	215.2 ± 64.44	428.2 ± 171.0	337.8 ± 87.49
Total conjugated BAs%	45.73 ± 2.13	67.72 ± 1.35***	62.14 ± 1.97#
Total unconjugated BAs (ng/mg liver tissue)	259.8 ± 90.17	191.6 ± 74.39	188.7 ± 43.11
Total unconjugated BAs%	54.27 ± 2.13	32.28 ± 1.35***	37.86 ± 1.97#
Conjugated BAs/unconjugated BAs	0.86 ± 0.07	2.13 ± 0.12***	1.69 ± 0.13#
Total T-con BAs (ng/mg liver tissue)	193.2 ± 57.05	362.4 ± 136.3	296.2 ± 72.53
Total T-con BAs%	41.38 ± 2.05	59.49 ± 1.04	55.40 ± 1.69
Total G-con BAs (ng/mg liver tissue)	21.99 ± 7.48	65.81 ± 34.84	41.55 ± 16.02
Total G-con BAs%	4.35 ± 0.32	8.23 ± 0.95**	6.75 ± 0.83
T-con/G-con BA	9.75 ± 0.84	7.85 ± 0.78	8.84 ± 0.77
CA/DCA	5.06 ± 1.06	4.48 ± 1.02	4.07 ± 1.12
TDCA/DCA	12.04 ± 3.09	28.71 ± 14.56	26.65 ± 9.29
TDCA/CA	2.26 ± 0.31	7.60 ± 2.87	19.93 ± 9.99
TCA/CA	27.42 ± 4.71	107.1 ± 65.19	178.0 ± 95.20
GCA/TCA	0.12 ± 0.01	0.17 ± 0.03	0.15 ± 0.02
GDCA/TDCA	0.01 ± 0.002	0.004 ± 0.0009	0.003 ± 0.0006

Note: All values were expressed as mean ± SEM. *p < 0.05, **p < 0.01, and ***p < 0.001, when NASH models (the HFD group) were compared to control groups (the NC group); #*p* < 0.05, when NASH models (the HFD group) were compared to NASH models treated with kaempferol (the HFD + KP group).

### Correlations between liver BA-relevant variables and clinical indices

**FIGURE 6:**
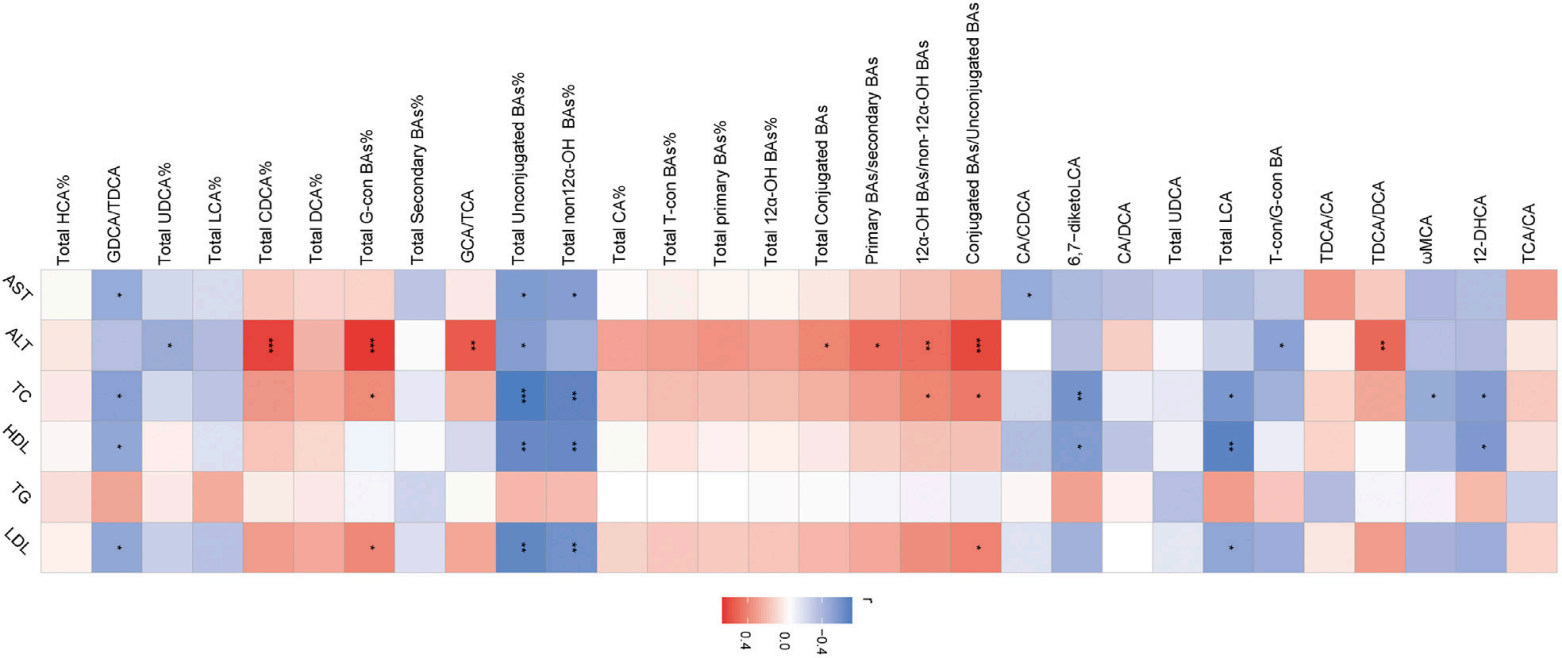
Heatmap of Spearman correlation coefficients between liver BAs and serum bio-chemical parameters from all samples in the three groups. n = 22, six samples for the control group, and eight samples for the other two groups. The gradient colors represent the correlation coefficients, with red color being more positive and blue color being more negative. *p < 0.05, **p < 0.01, and ***p < 0.001 (Spearman’s correlation with the *post hoc* correction using the Holm method).

The association of liver BAs and serum biochemical parameters was elucidated among three groups by Spearman correlation analysis. We found that most of BAs had a negative correlation with serum AST including CA/CDCA, total non-12α-OH BAs%, total unconjugated BAs%, and GDCA/TDCA. TDCA/DCA, conjugated BAs/unconjugated BAs, 12α-OH BAs/non-12α-OH BAs, primary BAs/secondary BAs, total conjugated BAs, GCA/TCA, total G-con BAs%, and total CDCA% had a positive correlation, and T-con/G-con BA, total unconjugated BAs%, and total UDCA% had a negative correlation with serum ALT. Conjugated BAs/unconjugated BAs, 12α-OH BAs/non-12α-OH BAs, and total G-con BA% had a positive correlation with serum TC, and 12-DHCA, total LCA, 6,7-diketoLCA, total non-12α-OH BAs%, total unconjugated BAs%, and GDCA/TDCA had a negative correlation with serum TC and HDL. Moreover, ωMCA had a negative correlation with serum TC. Conjugated BAs/unconjugated BAs and total G-con BAs% had a positive correlation, and total LCA, total non12α-OH BAs%, total unconjugated BAs%, and GDCA/TDCA had a negative correlation with serum LDL (Figure 6).

### Kaempferol induces the expression of BA synthase and transporters in the liver

**FIGURE 7:**
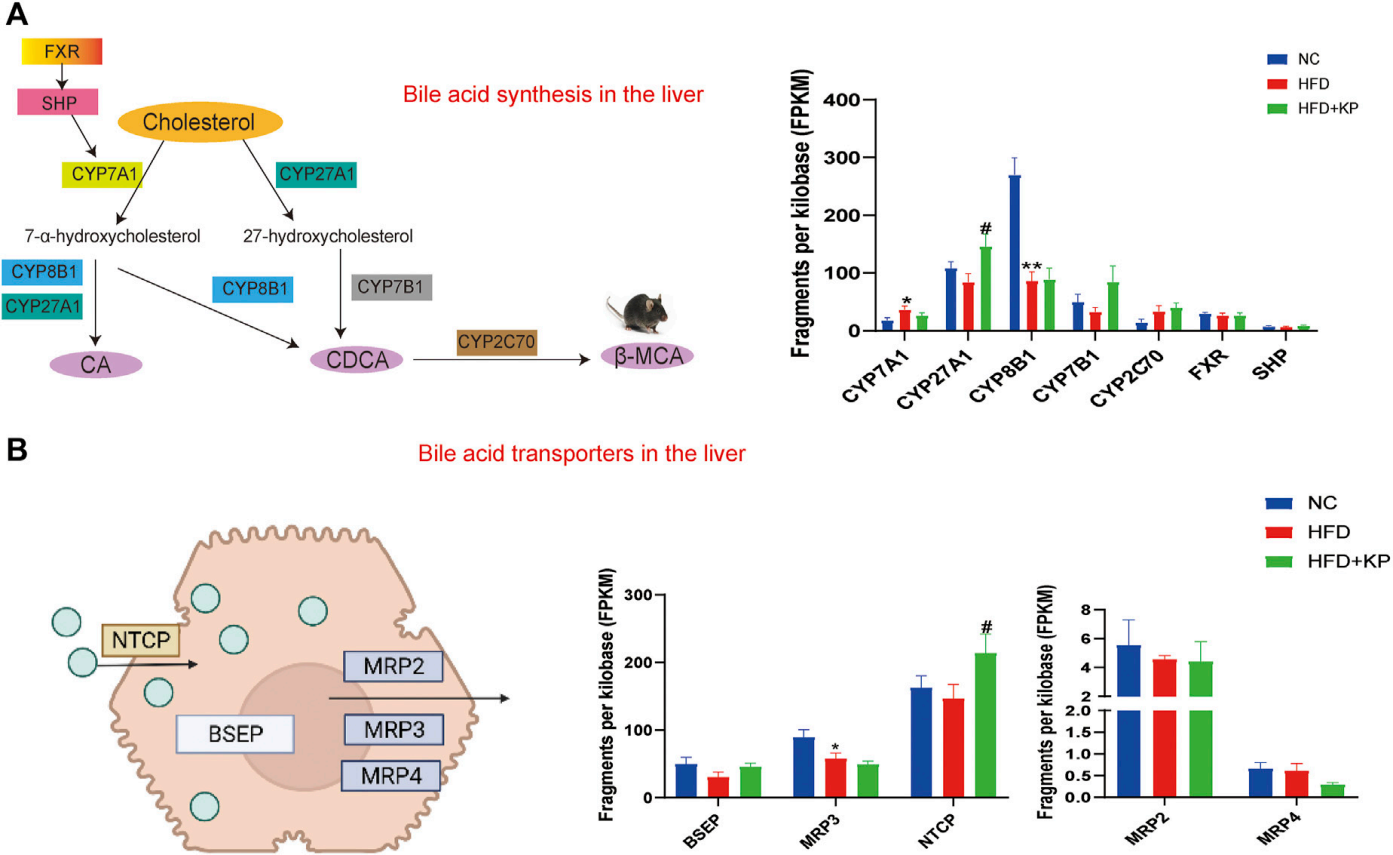
Kaempferol induces the expression of BA synthase and transporters in the liver. **(A)** Diagram and mRNA analysis of bile acid biosynthesis markers in the liver (n = 5 per group). **(B)** Diagram and mRNA analysis of bile acid transporters in the liver (n = 5 per group). All values were expressed as mean ± SEM. *p < 0.05 and **p < 0.01, when NASH models (the HFD group) were compared to control groups (the NC group); #*p* < 0.05, when NASH models (the HFD group) were compared to NASH models treated with kaempferol (the HFD + KP group). CYP7A1, cholesterol-7α-hydroxylase; CYP27A1, mitochondrial sterol 27-hydroxylase; CYP8B1, sterol 12α&hyphen; hydroxylase; CYP7B1, oxysterol 7α&hyphen ;hydroxylase; CYP2C70, cytochrome P450 family 2 subfamily c polypeptide 70; FXR, farnesoid X receptor; SHP, small heterodimer partner; BSEP, bile salt export pump; MRP2, multidrug resistance-related protein 2; MRP3, multidrug resistance-related protein 3, MRP4, multidrug resistance-related protein 4; NTCP, Na + -taurocholate cotransporting polypeptide.

In order to explore the serum and liver changes of BA levels among three groups after kaempferol intervention, we combined the results from transcriptomics (Lu et al., 2020) to detect the mRNA expression of BA synthase and transporter in the liver. Analysis of mRNA for enzymes in the two BA synthesis pathways is performed, including CYP7A1, CYP27A1, CYP8B1, oxysterol 7α‐hydroxylase (CYP7B1), cytochrome P450 family 2 subfamily c polypeptide 70 (CYP2C70), farnesoid X receptor (FXR), and small heterodimer partner (SHP). We found that compared with the NC group, the mRNA expression of CYP7A1 in the HFD group was significantly increased, and the mRNA expression of CYP8B1 was significantly decreased, while kaempferol significantly promoted the mRNA expression of CYP27A1 (Figure 7A). In the enterohepatic circulation, transporters play a key role in maintaining BA homeostasis. Thus, we simultaneously paid attention to the mRNA expression of BA transporters in the liver including the bile salt export pump (BSEP), multidrug resistance-related protein 3 (MRP3), Na + taurocholate cotransporting polypeptide (NTCP), MRP2, and MRP4. We found that in the HFD group, the mRNA expression of MRP3 was significantly decreased, and at the same time, kaempferol could significantly upregulate the mRNA expression of NTCP (Figure 7B).

## Discussion

NASH is the common decisive stage for non-alcoholic fatty liver disease (NAFLD) and also a pathogenic factor for end-stage liver disease. BAs play pleiotropic roles in fat absorption and metabolism, and BA disturbances are associated with the pathogenesis of NASH (Jiao et al., 2022). Our previous study had already showed that kaempferol reduced the levels of ALT, LDL, and TC in the serum and TG and lipid droplets and inflammatory cell infiltration in the liver (Lu et al., 2020). At the same time, we also confirmed that kaempferol can effectively improve fatty degeneration caused by Phosphatidic acid/Oleic acid (PA/OA) *in vitro* (Xiang et al., 2021). In this study, we further compared serum and liver BA profiles in normal, NASH, and kaempferol-treated NASH mice. Compared with HFD groups, the downward trend of serum levels of βMCA, UDCA, and ωMCA and liver levels of 12-DHCA and ωMCA, as well as the upward trend of serum levels of TDCA, THDCA, and TUDCA could be blocked by kaempferol. To the best of our knowledge, this is the first time to clarify the possible mechanism of kaempferol improving NASH from the perspective of BAs.

### Kaempferol improves NASH by regulating the homeostasis of BA pool

Bile acids play an important physiological role not only in promoting intestinal absorption and nutrient transport but also in maintaining systemic lipid homeostasis. First, we analyzed the level of BAs among three groups.

MCA, as an important part of the mouse bile acid pool, was found to alleviate and inhibit lipid accumulation by inhibiting the absorption of cholesterol (Wang et al., 2003). Similarly, we found that the contents of βMCA and ωMCA in the serum of NASH mice were decreased, and the ωMCA level in the liver was decreased, and kaempferol can regulate this situation. This indicates that kaempferol may alleviate NASH by increasing the level of MCA to regulate lipid metabolism.

Excessive accumulation of secondary acids will destroy the late homeostasis of the BA pool. Higher levels of TDCA may indicate hepatic overproduction of CA, possibly by abnormally affecting hepatic BA-mediated signaling (Rivera-Andrade et al., 2022). Similarly, this study also found the same phenomenon, and kaempferol can reduce the level of TDCA to regulate BA homeostasis.

In this study, we also found that the level of UDCA in NASH mice was significantly lower than that in control mice, and kaempferol could increase the level of UDCA. Xie et al. (2022) found that Exe-UBC, a derivative of UDCA, can activate the expression level of sirtuin-1 (SIRT1) and inhibit the expression of peroxisome proliferator-activated receptor-γ coactivator-1β (PGC-1β) and peroxisome proliferator-activated receptor gamma (PPAR-γ), thereby reducing lipid deposition.

Tang et al. (2019) found that THDCA was decreased in the serum of a rat NAFLD model. However, in this study, the level of THDCA was found to be elevated in NASH mice, which may be related to the different animal species.

TUDCA can treat cholestatic diseases mainly by increasing the bile flow and BA secretion, attributed to choleretic and cytoprotective effects on hepatocytes (Paumgartner and Beuers, 2002), and its mechanism is mainly related to alleviating ERS (Latif et al., 2022). However, in this study, the level of TUDCA was found to be elevated in NASH mice.

Chanussot et al. (1992) found that DHCA can inhibit the secretion of phospholipids and cholesterol to achieve the effect of protecting the liver. Similar results were found in the present study. Kaempferol can relieve NASH by increasing the level of 12-DHCA and promoting the secretion of cholesterol.

Haeusler et al. (2016) found that obesity was positively associated with increased BA synthesis and 12α-hydroxylated BA (12α-OH BA), including CA and DCA. Similar results were obtained in the mouse liver in this study, finding that total CA%, total primary BAs, and primary BAs/secondary BAs were significantly elevated in NASH mice, and kaempferol was able to modulate this disorder, which indicates that kaempferol can alleviate NASH by reducing the oversynthesis of BA in the liver. In addition, Puri et al. (2018) had found that changes in circulating BA profiles were correlated with NAFLD disease severity. The ratio of conjugated primary BAs to unconjugated primary BAs was significantly increased in NASH patients. Haeusler et al. (2013) also found that the 12α-OH BA/non 12α-OH BA was positively correlated with insulin resistance (IR), and this result was also verified in this study, indicating that kaempferol may inhibit IR by regulating the ratio within BA resistance to alleviate NASH, which may be a new direction for future research.

### Kaempferol improves NASH by regulating bile acid-related nuclear receptors and cellular signaling pathways.

Combined with the results of RNA-seq, we found that the mRNAs of CYP7A1 were significantly increased and those of CYP8B1 were significantly decreased in NASH mice, which indicated that high-fat diet mainly affected the classic BA biosynthesis pathway (Chiang, 2004). As an important transcription factor of BA synthase and transporter, FXR is closely related to the balance of BA synthesis, influx, and efflux. In the liver, BA-induced FXR activation inhibits CYP7A1 by upregulating SHP (Halilbasic et al., 2013). Our experiments showed that in the liver, the levels of FXR and SHP mRNA did not change between the three groups. This indicates that the hepatic FXR signaling pathway is not inhibited, but the regulatory mechanism of the FXR signaling pathway can be further clarified by detecting FXR in the intestinal tissue in the later stage (Xue et al., 2021). This is consistent with an increase in total CA% and total CDCA%. In terms of BA transport, BAs returned to the liver from the portal vein are absorbed by NTCP, and BSEP and MRP2 are the main transporters responsible for the excretion of monovalent BAs into bile through the tubular membrane (Trauner and Boyer, 2003). Our study found that kaempferol can increase the expression of NTCP. This indicates that kaempferol may modulate the BA pool by enhancing BA transport, thereby alleviating NASH. In the presence of hepatocyte BA overload, alternative basolateral BA transporters, such as MRP3 and MRP4, can transport excess BA to the systemic circulation. However, the total CA level in the serum did not change, which may be due to the upregulation of BSEP and MRP2.

### Limitations

Current literature reports on the association between BA components and NASH are inconsistent (Dasarathy et al., 2011; Lake et al., 2013; Jiao et al., 2018; Puri et al., 2018). It is difficult and speculative to interpret only by the data available in the present study. On the one hand, our analysis of CYP8B1, FXR, NTCP, BESP, MRP2, MRP3 and MRP4 only focused on mRNA expression and need to be further verified in the future research in the protein expression. This is mainly because the sampling of BAs in this study only passes through the blood and liver, and the BA in the blood has been separated from the enterohepatic circulation. Therefore, in the future, the detection of BA in the intestine can further explain the enterohepatic circulation and the important regulatory role of kaempferol in the intestine.

## Conclusion

In summary, our study suggests that 11 serum BAs and 3 hepatic BAs may be potential markers for kaempferol to improve NASH. Our findings provided more favorable evidence for an improved therapeutic relationship of kaempferol and NASH through regulating BA profiles.

## Data Availability

The datasets presented in this study can be found in online repositories. The names of the repository/repositories and accession number(s) can be found below: NCBI GEO, GSE145665.
