# Supplementary material for: Kaempferol attenuates nonalcoholic steatohepatitis by regulating serum and liver bile acid metabolism
Source: Front Pharmacol. 2022 Sep 29;13:946360. doi: 10.3389/fphar.2022.946360 (PMC9557056; doi:10.3389/fphar.2022.946360)
Supplement: Supplementary file 1 [file DataSheet1.pdf]

Kaempferol attenuates nonalcoholic steatohepatitis by regulating serum and liver bile acid metabolism

Yifei Lu[1,3], Mingmei Shao[1,4], Caiyun Zhang[1], Hongjiao Xiang[1], Junmin Wang[1], Tao Wu[1,2*] and Guang Ji[2*]

1 Institute of Interdisciplinary Integrative Medicine Research, Shanghai University of Traditional Chinese Medicine, Shanghai, China.

2 Institute of Digestive Disease, Longhua Hospital, Shanghai University of Traditional Chinese Medicine, Shanghai, China.

3 Yueyang Hospital of Integrated Traditional Chinese and Western Medicine, Shanghai University of Traditional Chinese Medicine, Shanghai, China.

4 Baoshan District Hospital of Intergrated Traditional Chinese and Western Medicine, Shanghai, China.

*co-corresponding author.

Correspondence to: Prof. Tao Wu, PhD. Institute of Interdisciplinary Integrative Medicine Research, Shanghai University of Traditional Chinese Medicine, Cailun Road 1200, Shanghai 201203, China. Email: tw827@shutcm.edu.cn or wutao001827@163.com; Prof. Guang Ji, PhD. Institute of Digestive Disease, Longhua Hospital, Shanghai University of Traditional Chinese Medicine, South Wanping Road 725, Shanghai 200032, China. Email: jg@shutcm.edu.cn or jiliver@vip.sina.com.

SUPPLYMENTARY MATERIALS

Supplymentary Methods

Sample Preparation and UPLC-MSMS analysis

Figure S1. Comparison of serum BA among three groups. **(A)** Heatmap of all serum BAs among three groups. **(B)** Percent of primary BAs and secondary BAs among groups.

Figure S2. Comparison of liver BA among three groups. **(A)** Heatmap of all liver BAs among three groups. **(B)** Percent of primary BAs and secondary BAs among

groups.

Table S1. Equation of Bile acids sums, ratios or percent.

Table S2. Full name of 29 Bile acids.

Supplymentary Methods

Sample Preparation and UPLC-MSMS analysis

Chemicals and Reagents

All of the 400 standards including almost 45 bile acids (BAs) were obtained from Sigma-Aldrich (St. Louis, MO, USA), Steraloids Inc. (Newport, RI, USA) and TRC Chemicals (Toronto, ON, Canada). All the standards were accurately weighed and prepared in water, methanol, sodium hydroxide solution, or hydrochloric acid solution to obtain individual stock solution at 5.0 mg/mL. Appropriate amount of each stock solution was mixed to create stock calibration solutions. Analytical-grade formic acid was obtained from Sigma-Aldrich (St. Louis, MO, USA). Methanol (Optima LC-MS), acetonitrile (Optima LC-MS), and isopropanol (Optima LC-MS) were purchased from Thermo-Fisher Scientific (FairLawn, NJ, USA). Ultrapure water was obtained from a Mill-Q Reference system (Millipore, Billerica, MA, USA).

Sample Preparation

First, 50 mg of liver and 50 μL of serum were processed according to our previous sample preparation and derivatization protocols based on the method previously published with minor modifications (Int J Cancer, 2016, 139, 1764-1775; Journal of Proteome Research2021, 20, 8, 3814–3825). Then 25μL of serum was added to a 96-well plate and transferred to the Biomek 4000 workstation (Beckman Coulter, Inc., Brea, California, USA). Ice cold methanol with partial internal standards was automatically added to each sample and vortexed vigorously for 5 min. The plate was centrifuged at 4000g for 30 min (Allegra X-15R, Beckman Coulter, Inc., Indianapolis, IN, USA). Then the plate was returned back to the workstation. 30μL of supernatant was transferred to a clean 96-well plate, and 20μL of freshly prepared derivative reagents was added to each well. The plate was sealed and the derivatization was carried out at 30°C for 60 min. After derivatization, 350μL of ice-cold 50% methanol solution was added to dilute the sample. Then the plate was stored at -20 ℃ for 20 min and followed by 4000g centrifugation at 4 °C for 30 min. 135μL of supernatant was transferred to a new 96-well plate with 15μL internal standards in each well. Serial dilutions of derivatized stock standards were added to the left wells. Finally, the plate

was sealed for LC-MS analysis.

Analytical Quality Control Procedures

The rapid turnover of many intracellular metabolites makes immediate metabolism quenching necessary. The extraction solvents are stored in -20 ℃ freezer overnight and added to the samples immediately after the samples were thawed. All the prepared samples should be analyzed within 48 hours. Reproducible and accurate results are critical for quantitative metabolomics. In order to acquire reliable and accurate results, three types of quality control samples i.e., test mixtures, stable isotope-labelled internal standards, and quality controls are routinely used in the metabolomics platform. In addition to the quality controls, solvent blank samples are also used to obtain optimal instrument performance. The Metabolite Array kit includes a test mixture comprising all of the metabolite reference standards. The test samples were analyzed at the beginning of each batch to ensure that the instruments were performing within laboratory specifications (retention time stability, chromatographic peak shape, and peak signal intensity). The retention time shift for a batch of 84 samples should be within 4 sec. Internal standards were added to the test samples in order to control analytical variations during the whole process of sample preparation and analysis. Reagent blank samples are a mixture of solvents used for sample preparation and are commonly processed using the same procedures as the samples to be analyzed. The reagent blanks serve as a useful alert to systematic contamination. As the reagent blanks consist of high purity solvents and are analyzed using the same methods as the study samples, they are also used to wash the column and remove cumulative matrix effects throughout the study. The calibrators consist of a zero sample, and a series of ten concentrations covering the expected range for the metabolites present in the specific biological samples. LLOQ and ULOQ are the lowest and highest concentration of the standard curve that can be measured with acceptable accuracy and precision.

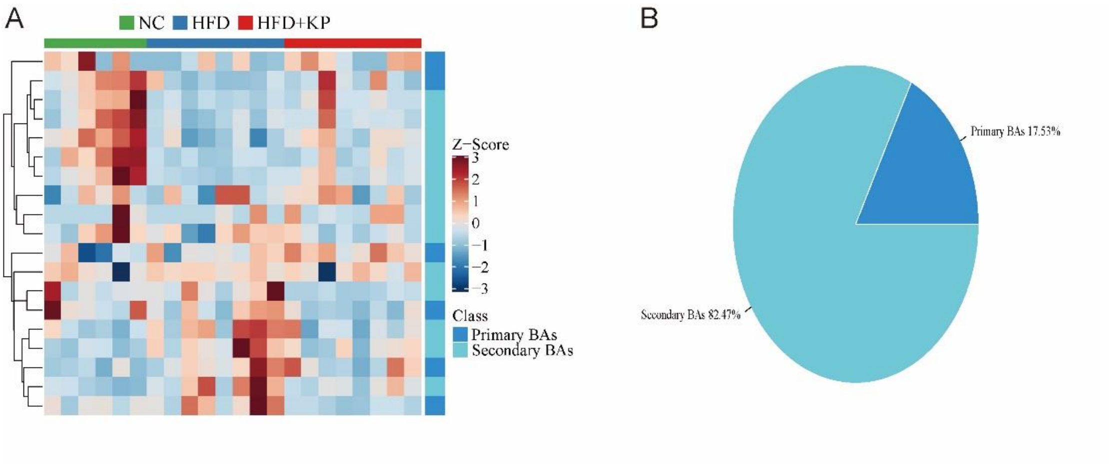

Figure S1. Comparison of serum BA among three groups. **(A)** Heatmap of all serum BAs among three groups. **(B)** Percent of primary BAs and secondary BAs among groups.

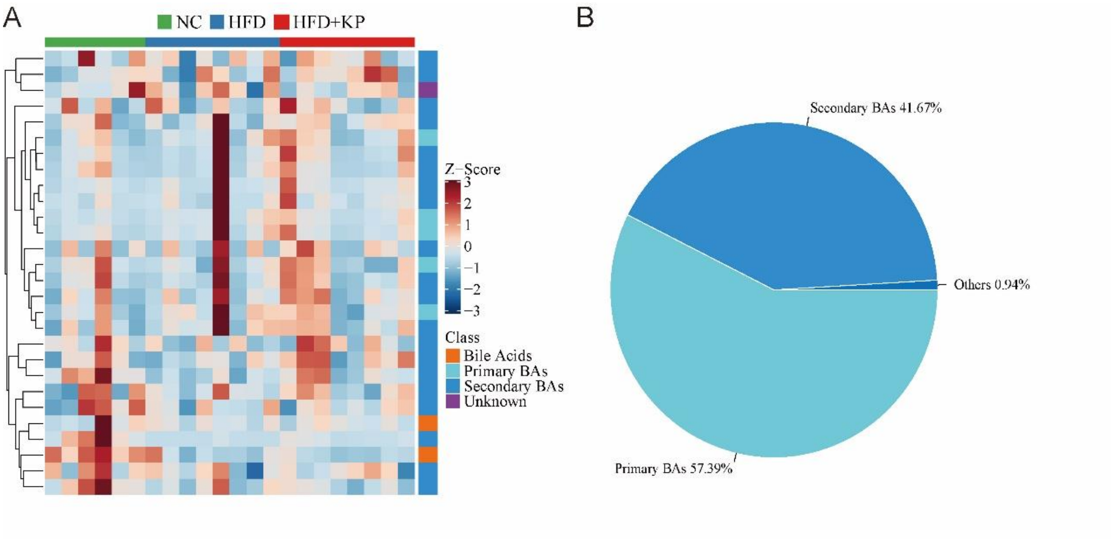

Figure S2. Comparison of liver BA among three groups. **(A)** Heatmap of all liver BAs among three groups. **(B)** Percent of primary BAs and secondary BAs among groups.

Table S1. Equation of Bile acids sums, ratios or percent.

NO	Ratios/Percent	Equation
1	Total CA	(CA+TCA+GCA+apoCA)
2	Total CA%	(CA+TCA+GCA+apoCA)/TBA*100%
3	Total CDCA	(CDCA+TCDCA)
4	Total CDCA%	(CDCA+TCDCA)/TBA*100%
5	CA/CDCA	CA/CDCA
6	Total UDCA	UDCA
7	Total UDCA%	UDCA/TBA*100%
8	Total DCA	(DCA+TDCA+GDCA+12-ketoLCA+norDCA)
9	Total DCA%	(DCA+TDCA+GDCA+12-ketoLCA+norDCA)/TBA*100%
10	Total LCA	(LCA+7-ketoLCA+6,7-DiketoLCA+7,12-DiketoLCA)
11	Total LCA%	(LCA+7-ketoLCA+6,7-DiketoLCA+7,12-DiketoLCA)/TBA*100%
12	Total HCA	GHCA
13	Total HCA%	GHCA/TBA*100%
14	Total Primary BAs	(CA+CDCA+GCA +TCA+TCDCA)
15	Total Primary BAs%	(CA+CDCA+GCA +TCA+TCDCA) /TBA*100%
16	Total secondary BAs	(12-ketoLCA, 7-ketoLCA, DCA, GDCA, GHCA, LCA, NorDCA, TDCA, UDCA)
17	Total secondary BAs%	(TBA- Primary BA)/TBA*100% OR (12-ketoLCA, 7-ketoLCA, DCA, GDCA, GHCA, LCA, NorDCA, TDCA, UDCA) /TBA*100%
18	Primary BAs / secondary BAs	(CA+CDCA+GCA +TCA+TCDCA)/ (TBA- Primary BA)
19	Total 12α-OH	(CA, DCA, GCA, GDCA, TCA, TDCA+norCA +norDCA)

	BAs	
20	Total 12α-OH BAs%	(CA, DCA, GCA, GDCA, TCA, TDCA+norCA+norDCA) /TBA*100%
21	Total non12α-OH BAs	(TBA- 12α-OH BAs) OR (12-ketoLCA, 7-ketoLCA, CDCA, GHCA, LCA, NorDCA, TCDCA, UDCA)
22	Total non12α-OH BAs%	(TBA- 12α-OH BAs) /TBA*100% OR (12-ketoLCA, 7-ketoLCA, CDCA, GHCA, LCA, NorDCA, TCDCA, UDCA) /TBA*100%
23	12α-OH BAs/non-12α-OH BAs	(CA, DCA,GCA, GDCA, TCA, TDCA+norCA +norDCA) / （TBA- 12α-OH BA）
24	Total conjugated BAs	(GCA, GDCA, GHCA, TCA, TCDCA, TDCA)
25	Total conjugated BAs%	(GCA, GDCA, GHCA, TCA, TCDCA, TDCA) /TBA*100%
26	Total unconjugated BAs	(TBA- Conjugated BAs) OR (12-ketoLCA, 7-ketoLCA, CA, CDCA, DCA, LCA, NorDCA and UDCA)
27	Total unconjugated BAs%	(TBA- Conjugated BAs)/TBA*100% OR (12-ketoLCA, 7-ketoLCA, CA, CDCA, DCA, LCA, NorDCA and UDCA) /TBA*100%
28	Conjugated BAs/ Unconjugated BAs	Conjugated BAs/ Unconjugated BAs
29	Total T-con BAs	(TCA, TCDCA, TDCA)

30	Total T-con BAs%	(TCA, TCDCA, TDCA) /TBA*100%
31	Total G-con BAs	(GCA, GDCA, GHCA)
32	Total G-con BAs%	(GCA, GDCA, GHCA) /TBA*100%
33	T-con/G-con BA	(TCA, TCDCA, TDCA, THCA, and TUDCA)/ (GCA, GCDCA, GDCA, GHCA, GLCA, GUDCA+)
34	CA/DCA	CA/DCA
35	TDCA/DCA	TDCA/DCA
36	TDCA/CA	TDCA/CA
37	TCA/CA	TCA/CA
38	GCA/TCA	GCA/TCA
39	GDCA/TDCA	GDCA/TDCA

Table S2. Full name of 29 Bile acids.

NO	Abbreviation	Full name
1	LCA	Lithocholic Acid
2	NorDCA	23-Nordeoxycholic Acid
3	6-ketoLCA	6-Ketolithocholic Acid
4	7-ketoLCA	7-Ketolithocholic Acid
5	7-ketoDCA	7-Ketodeoxycholic Acid
6	12-ketoLCA	12-Ketolithocholic Acid
7	ApoCA	Apocholic Acid
8	UDCA	Ursodeoxycholic Acid
9	HDCA	α-Hyodeoxycholic Acid
10	CDCA	Chenodeoxycholic Acid
11	DCA	Deoxycholic Acid
12	NorCA	Norcholic Acid

13	6,7-diketoLCA	6,7-diketolithocholic Acid
14	7,12-diketoLCA	7,12-diketolithocholic Acid
15	12-DHCA	12-Dehydrocholic Acid
16	3-DHCA	3-Dehydrocholic Acid
17	βCA	3β-Cholic Acid
18	βMCA	β-Muricholic Acid
19	ωMCA	ω-Muricholic Acid
20	CA	Cholic Acid
21	GDCA	Glycodeoxycholic Acid
22	GHCA	Glycohyocholate Acid
23	GCA	Glycocholic Acid
24	THDCA	Taurohyodeoxycholic Acid
25	TDCA	Taurodeoxycholic Acid
26	TCDCA	Taurochenodeoxycholic Acid
27	TωMCA	Tauro ω-muricholate Acid
28	TCA	Taurocholic Acid
29	CDCA-24G	Chenodeoxycholic Acid 24-Acyl-β-D-glucuronide Acid
